# Small extracellular vesicle miRNAs as biomarkers for predicting antitumor efficacy in lung adenocarcinoma treated with chemotherapy and checkpoint blockade

**DOI:** 10.3389/fimmu.2025.1573043

**Published:** 2025-03-31

**Authors:** Si Sun, Fuchuang Zhang, Jiyang Zhang, Hui Yu, Zhihuang Hu, Xiaoya Xu, Xinmin Zhao, Sheng Chen, Yao Zhang, Baoning Nian, Ying Lin, Zhikuan Li, Zhenhua Wu, Bo Yu, Xianghua Wu, Huijie Wang, Xiaohua Hui, Dadong Zhang, Jialei Wang

**Affiliations:** ^1^ Department of Thoracic Medical Oncology, Fudan University Shanghai Cancer Center, Shanghai, China; ^2^ Department of Oncology, Shanghai Medical College, Fudan University, Shanghai, China; ^3^ Institute of Thoracic Oncology, Fudan University Shanghai Cancer Center, Shanghai, China; ^4^ Department of Clinical and Translational Medicine, 3D Medicines Inc., Shanghai, China

**Keywords:** sEVs, miRNA, immune checkpoint inhibitors, chemotherapy, lung cancer. sEV miRNAs predicting immunochemotherapy efficacy

## Abstract

Checkpoint blockade combined with chemotherapy has become an important treatment option for lung cancer patients in clinical settings. However, biomarkers that effectively identify true responders remain lacking. We assessed the potential of plasma small extracellular vesicle (sEV)-derived microRNAs (miRNAs) as biomarkers for predicting and identifying responders to combined immunochemotherapy. A total of 29 patients with lung adenocarcinoma who received pembrolizumab combined with pemetrexed and carboplatin were enrolled. The efficacy evaluation revealed that 24 patients obtained durable clinical benefits from combined immunochemotherapy, and the rest experienced disease progression. Using unsupervised hierarchical clustering, 56 differentially expressed miRNAs (DEMs) were identified between responders and nonresponders. Efficacy prediction models incorporating a combination of sEV miRNAs were established and showed good performance (area under the curve (AUC) > 0.9). In addition, we found that miR-96-5p and miR-6815-5p were notably downregulated in the nonresponder group, while miR-99b-3p, miR-100-5p, miR-193a-5p, and miR-320d were upregulated. These findings were further confirmed by clinical imaging. sEV miRNAs derived from patients with lung cancer showed promise for identifying true responders to combined immunochemotherapy.

## Graphical Abstract

Proposed model of sEV miRNAs as a promising non-invasive tool for predicting true responders of lung adenocarcinoma patients receiving immunotherapy combined with chemotherapy.

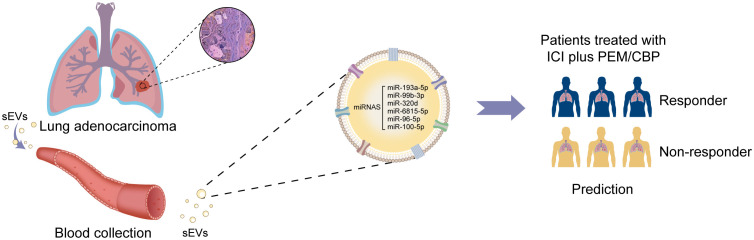

## 1 Introduction

Lung cancer has become a major threat to people’s health (1). In recent years, immunotherapy combined with chemotherapy has become an important lung cancer intervention. Tumor immunotherapy with immune checkpoint inhibitors represents a major breakthrough in lung cancer treatment. Pembrolizumab, as a programmed cell death-1 (PD-1) inhibitor, has been used in first- and second-line immunotherapy schemes for lung cancer (2). Pembrolizumab combined with chemotherapy drugs has shown a synergistic effect, prolonging the survival of lung cancer patients (3).

Programmed cell death ligand 1 (PD-L1) has been widely accepted as a predictive biomarker for immune check point inhibitors during immunotherapy. The PD-L1 expression level at the tumor cell surface may affect the efficacy of immune checkpoint inhibitors in patients with lung cancer (4, 5). The objective response rate to pembrolizumab-based immunotherapeutic strategies for the treatment of non-small-cell lung cancer has been found to be 19.4% in all patients (6). Even in the population with the highest PD-L1 expression (≥50%), the patient response rate was only 45.2% (6). Although more patients benefit from immunotherapy combined with chemotherapy, effective clinical efficacy evaluation biomarkers remain lacking. It is critical to explore predictive biomarkers for accurately screening responders to tumor immunotherapy combined with chemotherapy to reduce unnecessary medical resource waste, avoid potential side effects in nonresponders, and optimize the therapeutic efficacy of immunotherapy combined with chemotherapy.

Small extracellular vesicles (sEVs) are biologically active vesicles secreted by multivesicular bodies. sEVs are generally 30–150 nm in diameter and have a lipid bilayer membrane. The contents of sEVs, such as lipids, proteins, and nucleic acids play critical roles in intercellular communication. The stable characteristics and easily availability of sEVs make them a promising alternative approach for precision cancer medicine, especially early tumor screening, diagnosis, and treatment. Moreover, sEVs play an important role in tumorigenesis, metastasis, deterioration, and immune escape (7, 8). Studies have shown that tumor-delivered sEVs interact with stromal cells in the tumor microenvironment (9). Information transmission among tumor cells, sEVs, and the tumor microenvironment determines the development and malignant degree of tumors (10). Small RNAs in sEVs, including microRNAs (miRNAs), have been suggested as potential biomarkers for various cancers (11–14). However, whether sEV miRNAs can be used as predictive biomarkers to predict the efficacy of immunocombined chemotherapy remains unclear.

In this study, differentially expressed miRNAs (DEMs) in sEVs were analyzed by small RNA sequencing in patients with advanced or metastatic lung adenocarcinoma treated with immunotherapy combined with chemotherapy. Numerous combinations of sEV miRNAs were modeled, and efficient models containing 2 or 3 sEV miRNA combinations were identified for identifying true response populations and showed better performance (AUC >0.9). Moreover, the six sEV miRNAs in the least absolute shrinkage and selection operator (LASSO) model could be used to predict the prognosis of patients in the responder and nonresponder groups. The risk score of sEV miRNAs showed a correlation with tumor size changes. Our findings indicate that sEV miRNAs could be novel predictive biomarkers for responders to combination of immunotherapy with chemotherapy.

## 2 Materials and methods

### 2.1 Study approval

Blood samples were obtained from Fudan University Shanghai Cancer Center (FUSCC). This study was approved by the Ethics Committee of FUSCC (2004216–20–2005) and registered at ChiCTR (https://www.chictr.org.cn/) with registration number ChiCTR2000040854. All patients participating in this study signed informed medical consent. The analyses and experiments involving human blood samples were performed strictly in accordance with the regulations of the Declaration of Helsinki and Good Clinical Practice.

### 2.2 Patients and sample collection

Sixty patients with advanced NSCLC diagnosed as EGFR/ALK negative were recruited. The patients’ clinical characteristics are presented in **Supplementary Table 1**. After further pathological identification, 19 patients with non-lung adenocarcinoma were excluded (**Supplementary Figure 1**), and 29 patients who received the Pembro/Pem/Plat regimen were included in the final analysis (**Supplementary Figure 1**). Based on the RECIST 1.1 assessment, 14, 10, and 5 patients were included in the PR, SD, and PD groups, respectively (**Supplementary Figure 1**). Blood samples were collected from the 29 patients before treatment with immunochemotherapy and used for sEV extraction and subsequent analysis.

### 2.3 sEV isolation

sEVs were isolated as previously described (15). In brief, plasma samples were centrifuged (12,000 g, 4°C, 10 min) after incubation in a water bath (37°C, 5 min); 0.45μm filters were used to filter the collected supernatants. After subsequent centrifugation (12,000 g, 4°C, 5 min), 0.22-μm tube filters were used for filtration. The supernatant was collected and transferred to new tubes after further centrifugation (12,000 g, 4°C, 5 min); 25% of the volume of sEV isolation reagent (L3525; 3DMed, Shanghai) was then added to the supernatants. After gentle mixing and incubation (4°C, 30 min), the mixtures were centrifuged (4700 g, 4°C, 30 min) and the pellets containing sEVs were resuspended in phosphate-buffered saline (PBS).

### 2.4 NTA

The size and number of sEVs were characterized and tracked with the Nanosight NS 300 system (NanoSight Technology, Malvern). PBS was used to resuspend sEVs at a concentration of 5 μg/mL. The final concentration of sEVs was further diluted to 20–100 objects/frame. The sEV samples were added into sample chambers and were configured using a 488-nm laser under a scientific complementary metal-oxide semiconductor camera. Nanoparticle tracking data was analyzed with NTA analytical software (version 2.3), using the video tracks of each sample.

### 2.5 TEM

After resuspension in PBS, sEV samples were fixed with a paraformaldehyde (4%) solution. Then, sEVs were transferred to electron microscopy grids coated with carbon. After washing twice with PBS, the sEV samples were further washed with PBS containing glycine and then incubated with PBS with 0.5% BSA (10 min). Uranyl acetate (2%) was used to stain the grids, and sEVs were analyzed and photographed with a Hitachi H-7650 transmission electron microscope.

### 2.6 Nanoview analysis

Plasma sEVs were detected with ExoView Tetraspanin Chips. Antibodies against CD63 (Alexa-647), CD81 (Alexa-555), and CD9 (Alexa-488) were prepared and incubated with sEVs on the chips for 16 h. Then, PBS with Tween (PBST) and PBS was used to wash the chips containing sEVs. After washing, ExoView Tetraspanin kits were used for the co-localization analysis of antibodies. Finally, an ExoView R100 Scanner was used to image and characterize plasma sEVs. The results were further analyzed with NanoViewer Software.

### 2.7 qRT-PCR

The first-strand cDNA was synthesized using HiScript III reverse transcriptase (Vazyme, R302) in a reaction system (4 μL extracted RNA, 8.5 μL RNase-free water, 4 μL 5 × HiScript III Buffer, 1 μL 10 mM dNTPs, 0.5 μL HiScript III reverse transcriptase, 1μL RNase inhibitor and 1 μL specific miRNA stem-loop RT primer) with an appropriate procedure at 12°C for 5 min, 42°C for 15 min, 85°C for 5 min and then held at 4°C.

For qRT-PCR, a 25 μL reaction containing 2 μL cDNA, 9 μL PCR buffer, 1.5 μL Taq (Vazyme, P132), 0.75 μL hydrolysis probe (10 μM), 1.25 μL forward primer (30μM) and 1.75 μL universal reverse primer (10 μM) was conducted with 1 cycle of 95°C for 5 min, 45 cycles of 95°C for 10 s and 60°C for 35 s using the ABI 7500 fast real-time PCR System. Data were analyzed using the ΔΔ CT method, and miR-451 was regarded as the reference gene since as previously reported (14, 15). The primer sequences used for qRT-PCR are shown in **Supplementary Table 2**.

### 2.8 miRNA isolation from the serum sEVs

sEV miRNAs were isolated using the miRNeasy Serum/Plasma Kit according to the manufacturer’s instructions. (217184, QIAGEN, Shanghai, China). The yield, distribution, and quality of miRNAs were analyzed using an Agilent 2100 bioanalyzer and a Small RNA kit (5067-1548, Agilent, USA).

### 2.9 Small RNA library preparation and sequencing

A total of 6 µl RNA per sample was used to construct the miRNA library using the NEBNext Multiplex Small RNA Library Prep Set for Illumina (E7300L, NEB, USA) according to the manufacturer’s protocol. In brief, the library was obtained by 3′ adaptor ligation, hybridized reverse transcription primers, 5′ adaptor ligation, reverse transcription, and PCR amplification. Then, the DNA libraries were purified using a NucleoSpin Gel and PCR Clean-up kit (740609. 50, MACHEREY–NAGEL, Germany). The quality and distribution of libraries were analyzed using an Agilent 2100 bioanalyzer and High Sensitivity DNA kit (5067-4626, Agilent, USA). The libraries were sequenced using an Illumina NovaSeq 6000 analyzer.

### 2.10 Bioinformatics analysis of small RNA sequencing data

The small RNA-seq 3’-adaptors were trimmed using Cutadapt (DOI:10.14806/ej.17.1.200). Post-trimming, all retained reads were aligned to the human genome (GRCh37) and annotated using Gencode (v25) (16) and miRBase (v21) (17). Sequence alignment and the quantification of miRNA counts were performed using the voom function in the limma (v3.40.6) package (18), with normalization conducted using the trimmed mean of M-values (TMM) function in the edgeR (v3.26.8) package (19). The miRNA expression level was then converted into log2-counts-per-million (log2CPM). Differential miRNA expression was analyzed using the edgeR package.

Pathway and Gene Ontology enrichment of the experimentally validated miRNAs was performed using mirPath (v.3.0) (20), which provided an EASE score and false discovery rates using Fisher’s exact test and unbiased empirical distribution. The survival (v3.2.3) package (https://CRAN.R-project.org/package=survival) in R was used for the PFS analyses.

### 2.11 Statistical analysis

The diagnostic models in this study were constructed using LASSO (21). We selected the DEMs between responder and nonresponder groups based on a stringent statistical threshold (P ≤ 0.05, 1.5-fold change, and mean log2 expression ≥ 5). Based on different combinations of either two or three miRNAs that were significantly upregulated or downregulated in the PR samples, DEMs and risk scores were generated using LASSO analysis via the glmnet (v4.0.2) package (21), and the best model were constructed using LASSO. To assess the predictive potential of sEVs miRNAs concentration for patients’ response to immunochemotherapy, we examined several combinations of two or three miRNAs from DEMs, as per miRNA-seq data. Each group of miRNA combinations resulted in a risk scoring model being formed using the LASSO method. The ROC curve’s AUC was computed, and several combinations of two or three miRNAs with an AUC value exceeding 0.9 underwent screening.

## 3 Results

### 3.1 Characteristics of the subgroups of responder and nonresponder

**Figure 1:**
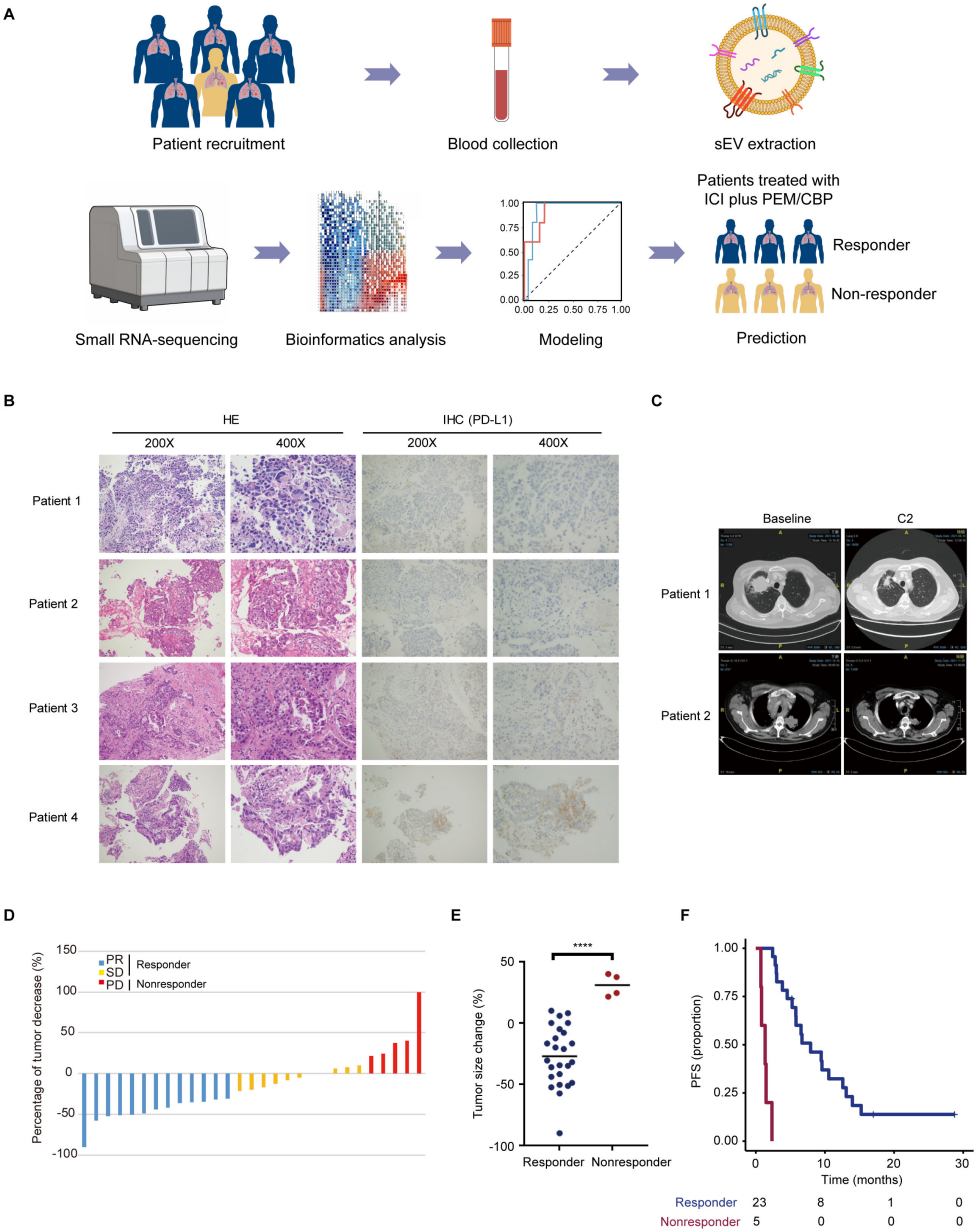
Characterization of the subgroups of responder and nonresponder. **(A)** Overview of the clinical workflow. Patients who signed informed consent were recruited. Blood samples from enrolled patients were collected and used for extraction and isolation of sEVs. After small RNA-sequencing, sEV miRNA was used for bioinformatics analysis. Following a differential analysis, 2-3 combinations of sEV miRNAs for modeling analysis. The sEV miRNA-based model can better predict the people who will benefit from the immune combination chemotherapy regimen. **(B)** Representative HE and PD-L1 IHC staining images of patients with lung adenocarcinoma. **(C)** Typical CT imaging features of the same patient before and after treatment. **(D, E)** Percentage of tumor changes in different groups ****p < 0.0001. **(F)** Patients diagnosed with nonresponder indicated a short survival.

The patient flow diagram is shown in **Supplementary Figure 1**, and the detailed clinical characteristics of the patients, including sex, age, smoking history, histology, ECOG PS, stage, drug, and efficacy evaluation are listed in **Supplementary Table 1**. In brief, 79% of the patients were male, and 66% had ever smoked (**Supplementary Table 1**). All patients were pathologically diagnosed with advanced or metastatic lung adenocarcinoma and received pembrolizumab combined with chemotherapy (pemetrexed plus carboplatin) (**Supplementary Table 1**). The clinical workflow is illustrated in **Figure 1A**. To better characterize the subgroups of responder and nonresponder, representative pathological and computed tomography (CT) images, tumor size changes, and prognostic differences were included in further analyses. Images of typical hematoxylin and eosin (HE), PD-L1 negative (patient 3 and patient 4), and PD-L1 positive (patient 1, PD-L1 15%; patient 2, PD-L1, 35%) staining of lung adenocarcinoma tumor tissue samples are shown in **Figure 1B**. Moreover, tumor size changes based on CT scan images are shown in **Figure 1C**. Patients were classified as PR, SD, and PD per their treatment response. The percentage of tumor changes in each enrolled patient is shown in **Figure 1D**. The percentage of tumor changes in the nonresponder group was significantly higher than that in the responder group (**Figure 1E**). Patients in the nonresponder group had a worse prognosis and reduced survival curves in comparison with those in the responder group (**Figure 1F**).

### 3.2 Representative of morphology and protein expression features of serum derived sEVs

**Figure 2:**
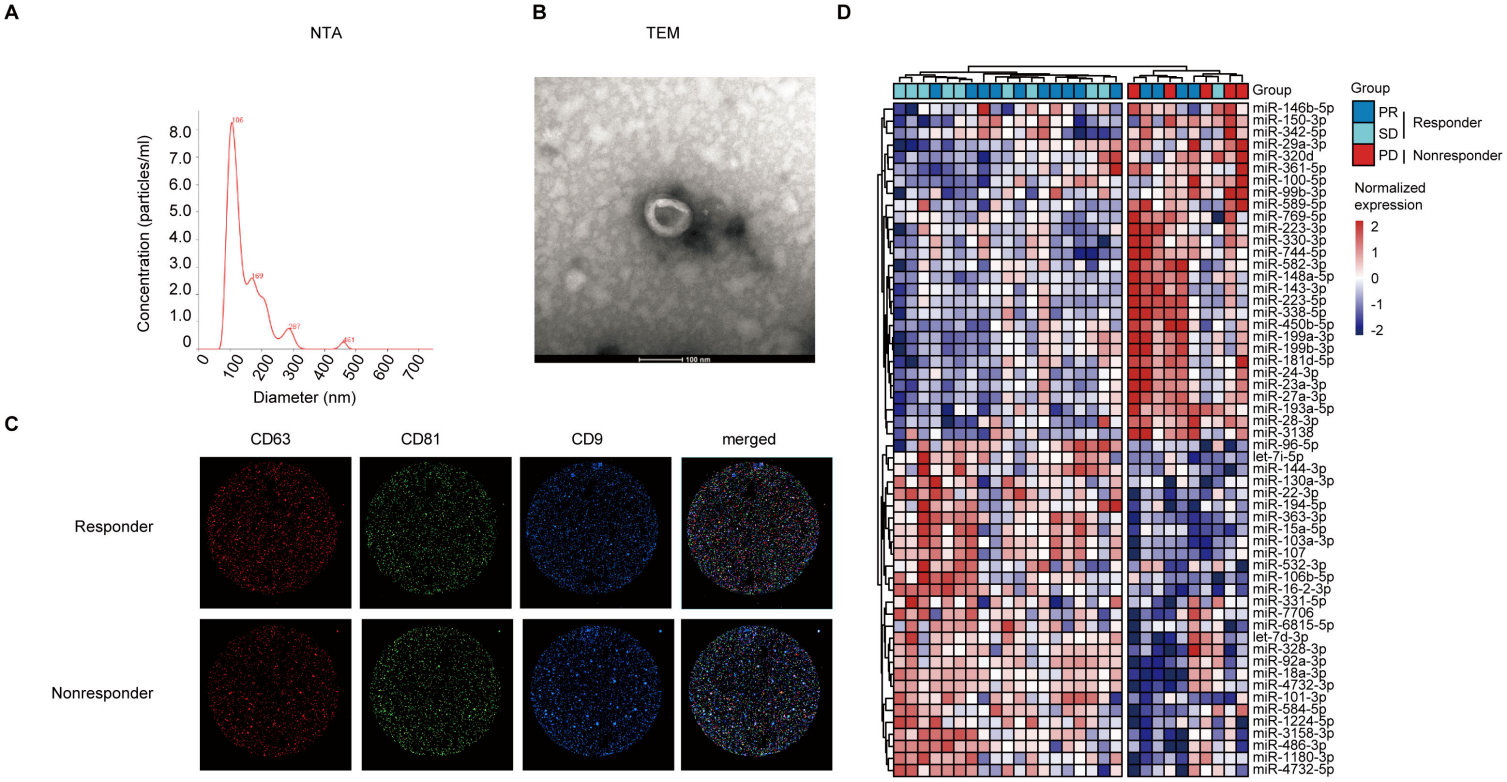
Representative of morphology and protein expression features of the serum-derived sEVs and sEV DEMs analysis. **(A)** The diameter distribution of sEVs based on NTA analysis. **(B)** TEM images of sEVs. Scale bar, 200 nm. **(C)** Single particle interferometric reflectance imaging analysis for the expression surface markers of serum sEVs. **(D)** The samples were clearly separated into two groups (responder vs. nonresponder) based on serum sEV DEMs with unsupervised hierarchical clustering analysis.

Nanoparticle tracking analysis (NTA) (**Figure 2A**), transmission electron microscopy (TEM) (**Figure 2B**), and immunofluorescence imaging (**Figure 2C**) were used to verify the purified sEVs with L3525 isolation method from lung adenocarcinoma patients. In brief, the diameters of sEVs are around 30–150 nm (**Figure 2A**). As shown in **Figure 2B**, the isolated sEVs demonstrated a “cup-shaped” or “dish-shaped” morphology. Moreover, an immunophenotypic analysis of single particle interferometric reflectance imaging, staining with CD63, CD81, and CD9 was used to further characterize the isolated sEVs (**Figure 2C**).

### 3.3 Serum-derived sEV miRNAs separated responder from nonresponder samples

The stable characteristics and easy availability of extracellular vesicles make them a promising alternative approach for precision cancer medicine, especially for early tumor screening, diagnosis, and treatment. Small RNA sequencing was used to explore the differentially expressed sEVs miRNAs between responders and nonresponders. Raw bioinformatic data from plasma-derived sEV miRNAs were normalized to counts per million. Preliminarily screening revealed 56 differentially expressed sEV miRNAs. As shown in **Figure 2D**, lung adenocarcinoma patients in nonresponder group could be distinguished from those in the responder groups based on the expression signature of sEV miRNAs.

### 3.4 Kyoto encyclopedia of genes and genomes and gene ontology analysis of the differentially expressed sEV miRNAs

**Figure 3:**
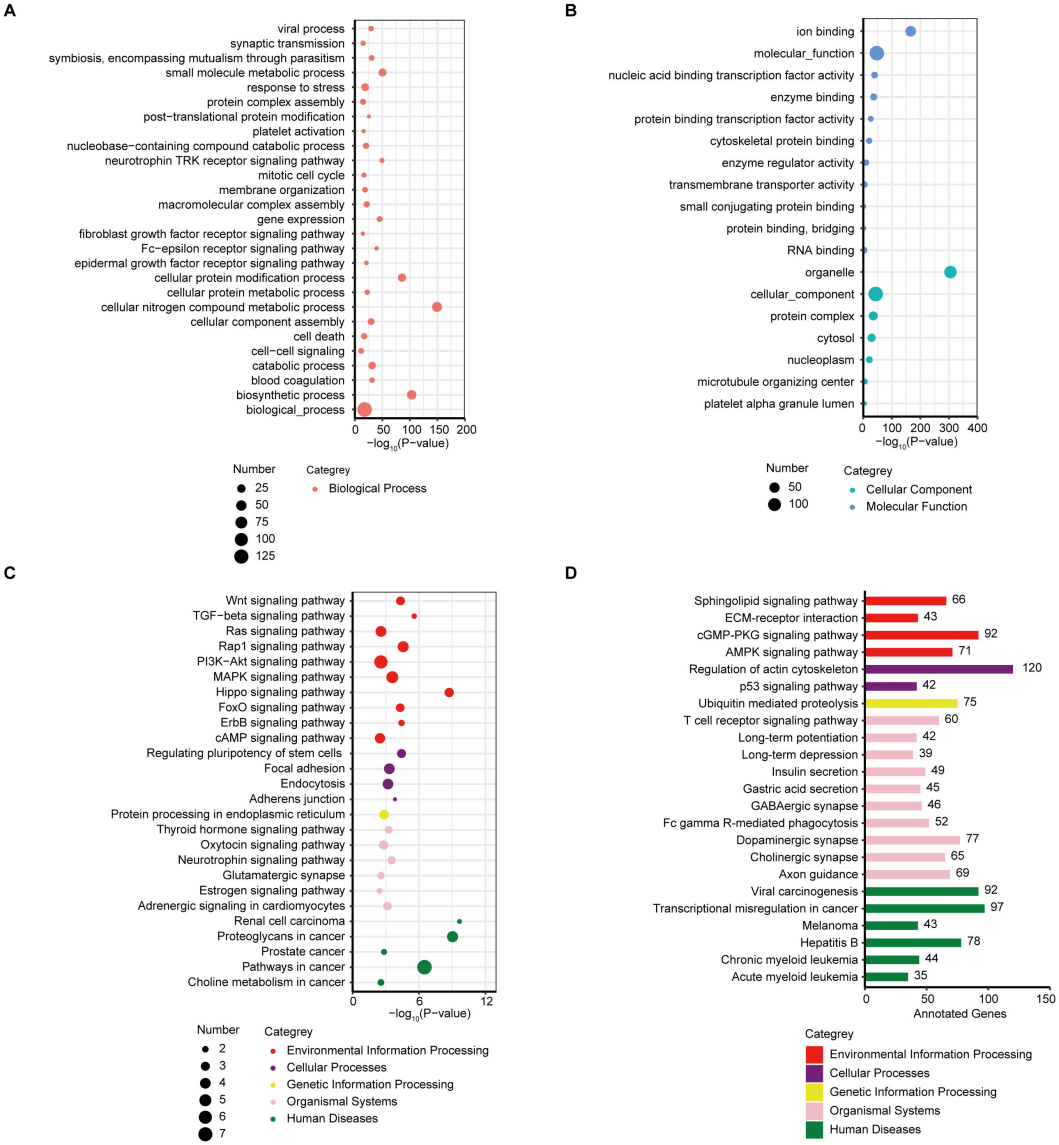
KEGG/GO analysis of the differentially expressed sEV miRNAs. **(A)** Bubble plot of enriched GO terms (biological process) of the target genes of the DEMs between responder and nonresponder groups. **(B)** Bubble plot of enriched GO terms (cellular component and molecular function) of the target genes of the DEMs between responder and nonresponder groups. **(C)** Bubble plot of enriched KEGG pathways of the target genes of the DEMs between responder and nonresponder groups. **(D)** Bar plot showing the number of DEM target genes in each of the KEGG pathways (target genes ≥ 35).

Next, bioinformatics assays based on the sEV miRNAs were performed to identify the potential regulatory mechanisms. The analysis of the differentially expressed plasma sEV miRNAs between patients in the nonresponder group and those in the responder groups indicated the involvement of multiple cancer related Kyoto Encyclopedia of Genes and Genomes (KEGG) pathways were involved. The KEGG database assays showed a strong enrichment for biological process related signaling pathways (**Figure 3A**), such as response to stress, small molecule metabolic process, and cell-cell signaling. Signaling pathways associated with cellular components and molecular function were found using bioinformatics miRNA target database analysis (**Figure 3B**). A strong enrichment for signaling pathways of environmental information processing, cellular processes, genetic information processing, organismal systems, and human diseases, was also observed (**Figure 3C**) in the DEM analysis. Furthermore, based on bioinformatic analysis of miRNA target databases, we analyzed the DEM targeting genes (n ≥ 35) within each KEGG pathway; the results are presented in **Figure 3D**.

### 3.5 Construction of risk score models for screening beneficiaries of immunochemotherapy

**Figure 4:**
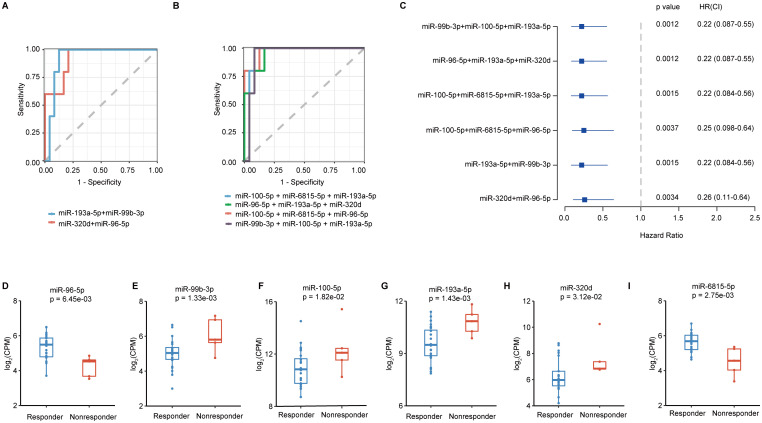
The construction of the risk score model for screening the beneficiaries of immunochemotherapy. **(A)** The best performance of ROC curves with two sEV miRNAs. **(B)** The best performance of ROC curves with three sEV miRNAs. **(C)** Hazard ratio calculations with different sEV miRNA combinations. **(D-I)** Analysis of the expression levels of miR-96-5p, miR-99b-3p, miR-100-5p, miR-193a-5p, miR-320d, and miR-6815-5p.

Combinations of different sEVs miRNAs were explored for the construction of a risk score model to better identify responders to immunotherapy combined with chemotherapy. Six groups of two or three sEV miRNA combinations with AUCs exceeding 0.9 (**Supplementary Tables 3**, **4**) were found. Among them, two groups contained two sEV miRNA combinations (miR-193a-5p, miR-99b-3p; miR-320d, miR-96-5p) (**Figure 4A**) and four groups contained three sEV miRNA combinations (miR-100-5p, miR-6815-5p, miR-193a-5p; miR-96-5p, miR-193a-5p, miR-320d; miR-100-5p, miR-6815-5p, miR-96-5p; miR-99b-3p, miR-100-5p, miR-193a-5p) (**Figure 4B**). Moreover, hazard models (**Figure 4C**) and progression-free survival (PFS) (**Supplementary Figures 2A–F**) were used to analyze the predictive role of the above six sEV miRNA combinations. Higher sEV miRNA combination scores indicated worse outcomes (**Figure 4C**, **Supplementary Figures 2A–F**). In addition, the expression of individual sEV miRNAs used to construct the risk score model were used for further analysis. Compared with the levels in patients in the responder groups, miR-96-5p and miR-6815-5p levels were significantly decreased in the nonresponder group (**Figures 4D, I**). However, the expression of miR-99b-3p, miR-100-5p, miR-193a-5p, and miR-320d showed opposite trends (**Figures 4E–H**). Thus, the risk score model with sEV miRNA combinations showed good performance in identifying responders to immunotherapy combined with chemotherapy.

### 3.6 Correlation between sEV miRNA expression and tumor change

The relationship between the percentage of tumor change and different sEVs miRNA panels was analyzed to explore the factors potentially influencing the risk score. A significant positive correlation was observed between the percentage of tumor change and the risk score of four different sEV miRNA panels: miR-320d and miR-96-5p (**Supplementary Figure 3A**), miR-100-5p, miR-6815-5p, or miR-96-5p (**Supplementary Figure 3C**), miR-100-5p, miR-6815-5p, and miR-193a-5p (**Supplementary Figure 3D**), and miR-96-5p, miR-193a-5p, and miR-320d (**Supplementary Figure 3E**). However, our results revealed no difference between the percentages of tumor change and risk score in the remaining sEV miRNA panels (miR-193a-5p and miR-99b-3p (**Supplementary Figure 3B**) and miR-99b-3p, miR-100-5p, and miR-193a-5p (**Supplementary Figure 3F**). The individual sEV miRNA used for model construction was then evaluated to further investigate the underlying reasons for the above findings. Our results indicated a strong positive correlation between miR-320d and the percentage of tumor change (**Supplementary Figure 4A**). The expression of miR-320d was obviously increased in patients in the PD group (**Supplementary Figure 4B**). However, the expression of miR-6815-5p was negatively correlated with the percentage of tumor change (**Supplementary Figure 4C**). Compared with the levels in the responder groups, the expression of miR-6815-5p was significantly decreased in the PD group (**Supplementary Figure 4D**). There were no significant associations between tumor change and miR-96-5p (**Supplementary Figure 4E**), miR-99b-3p (**Supplementary Figure 4F**), miR-100-5p (**Supplementary Figure 4G**), or miR-193a-5p (**Supplementary Figure 4H**).

### 3.7 Qualitative real-time polymerase chain reaction verification of selected plasma miRNAs

**Figure 5:**
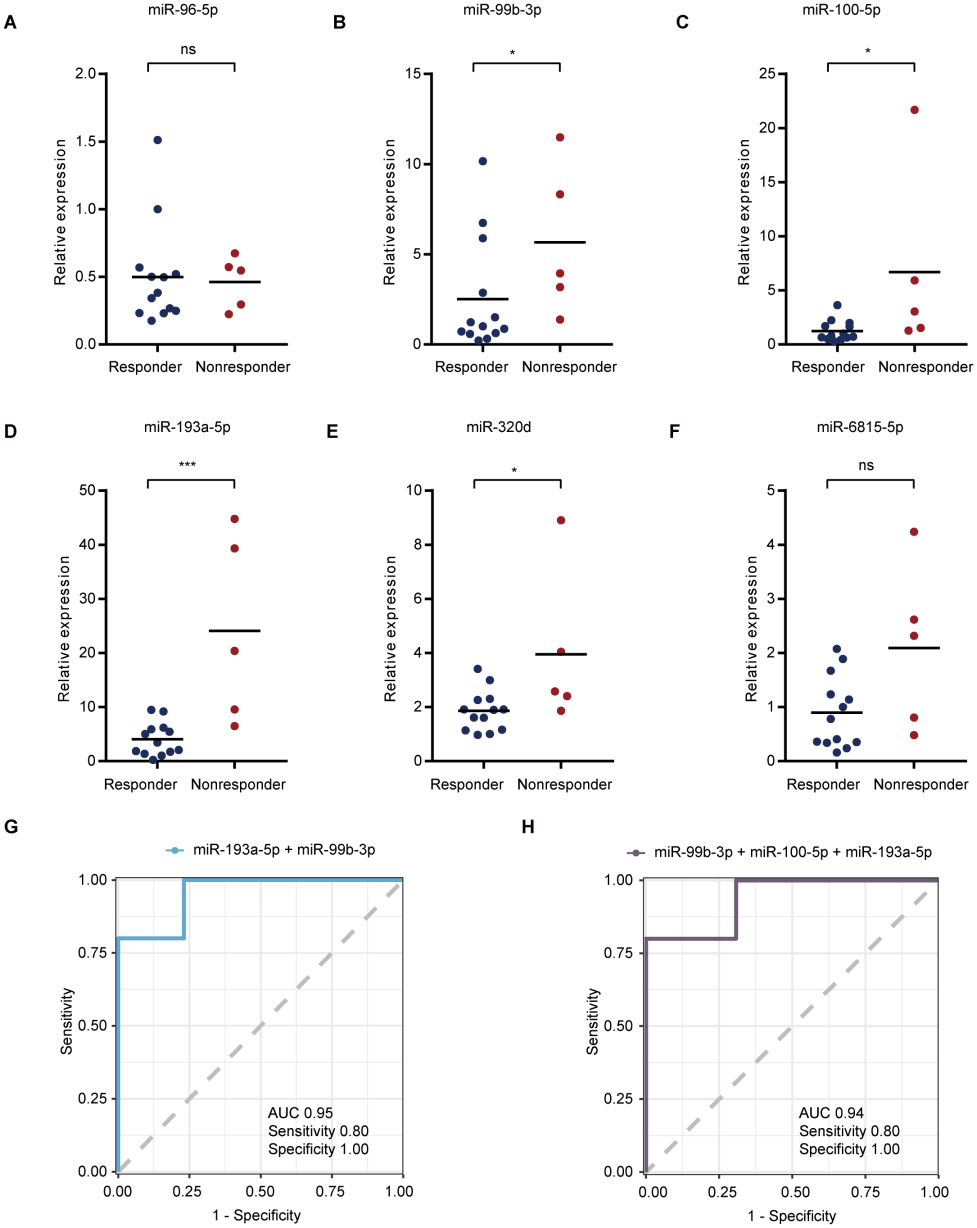
qRT-PCR verification of selected plasma miRNAs. **(A–F)** Validation of serum sEV miR-96-5p **(A)**, miR-99b-3p **(B)**, miR-100-5p **(C)**, miR-193a-5p **(D)**, miR-320d **(E)**, and miR-6815-5p **(F)** expression levels based on qRT-PCR analysis. **(G, H)** The best performance of ROC curves with two **(G)** or three **(H)** sEV miRNAs. *p < 0.05, ***p < 0.001, ns, not significant.

To further test whether the model can be verified using qualitative real-time polymerase chain reaction (qRT-PCR), we also verified sEV miRNA samples. Consistent with the above findings, miR-99b-3p (**Figure 5B**), miR-100-5p (**Figure 5C**), miR-193a-5p (**Figure 5D**), and miR-320d (**Figure 5E**) were highly expressed in the nonresponder group. However, the expression of miR-96-5p (**Figure 5A**) and miR-6815-5p (**Figure 5F**) in sEVs as verified by qRT-PCR was not significantly different between the two groups. In addition, miR-99b-3p, miR-100-5p, and miR-193a-5p, which had consistent verification results according to qRT-PCR, showed good identification performance in verifying the efficacy evaluation model based on two (**Figure 5G**) or three (**Figure 5H**) of the sEV miRNAs (**Supplementary Figures 5A–E**). These results provide a solid basis for further model development, as they demonstrate the reliability of serum sEV miRNAs by confirming their use through various detection techniques.

### 3.8 Predicted outcomes based on the risk score of different prediction models

**Figure 6:**
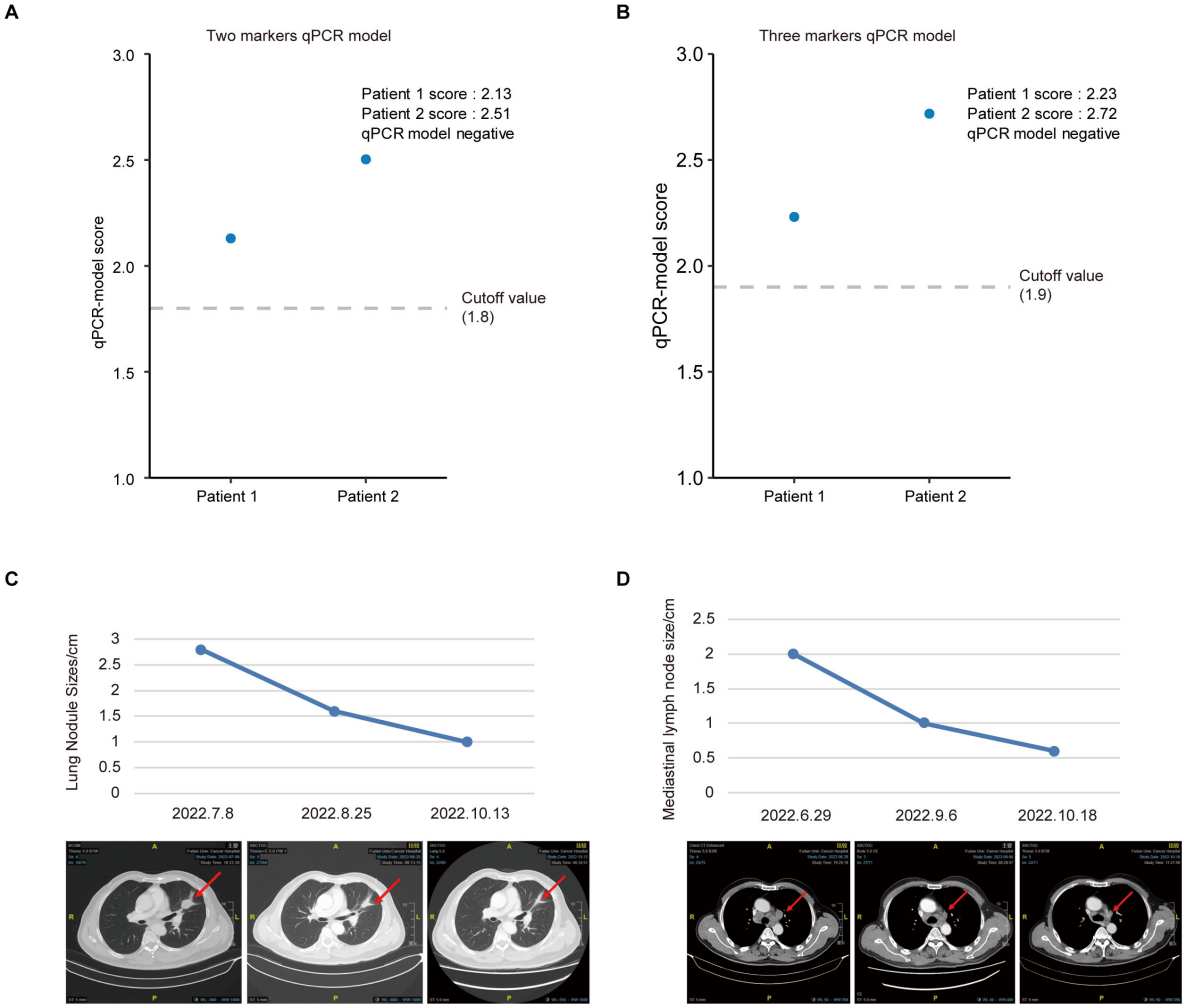
Predicted outcomes based on the risk score of different prediction model. **(A, B)** Examples of successful predictions of patient efficacy based on the two **(A)** or three **(B)** marker qRT-PCR model. **(C, D)** Examples of CT images and changes in the size of lung nodules **(C)** and mediastinal lymph nodes **(D)** after treatment with a combination of immunotherapy and chemotherapy.

To further test the models, we selected two random patients (Patient 1 and Patient 2) and used the qRT-PCR model scores to predict their efficacy. Interestingly, both the two (**Figure 6A**) and three (**Figure 6B**) marker qRT-PCR model demonstrated the potential to accurately and effectively predict the therapeutic response of patients undergoing immunocombination therapy. Moreover, imaging-based analysis also verified the accuracy of the model-based efficacy assessment (**Figures 6C, D**). These findings provide compelling evidence for the utility of an identification model in assessing the efficacy of immunotherapy with sEV miRNA combinations.

## 4 Discussion

Currently, the primary challenge of checkpoint blockade combined with chemotherapy is the lack of non-invasive and effective approaches for identifying true responders. It is widely accepted that blood sEVs play a critical role during the entire process of tumor progression. Cancer-specific information, such as miRNA and proteins, can be carried and detected in sEVs. The unique properties of tumor-derived sEV miRNAs make them ideal candidates for profiling tumor characteristics and screening responders to checkpoint blockade combined with chemotherapy. However, little is known about the potential value of plasma sEV miRNAs that can be used to predict whether patients with lung adenocarcinoma can benefit from immunochemotherapy.

In this study, we identified six sEV miRNAs (miR-193a-5p, miR-99b-3p, miR-320d, miR-6815-5p, miR-96-5p, and miR-100-5p) that showed significant differences in expression between responders and nonresponders. miR-96-5p and miR-6815-5p were downregulated in the nonresponder group and correlated with unfavorable responses to checkpoint blockade combined with chemotherapy. However, miR-99b-3p, miR-320d, miR-100-5p, and miR-193a-5p were obviously upregulated in nonresponders. Our results indicate that lung adenocarcinoma patients with low expression of sEV miR-99b-3p, miR-320d, miR-100-5p, and miR-193a-5p could benefit from checkpoint blockade combined with chemotherapy. Therefore, sEV miRNAs are promising biomarkers for effectively and accurately identifying true responders to immunochemotherapy.

The upregulation of miR-99b-3p, miR-320d, miR-100-5p, and miR-193a-5p in non-responders may be attributed to several potential mechanisms, which can inform the development of targeted therapies or combination strategies to improve treatment outcomes in lung adenocarcinoma. MiR-99b-3p has been reported to play a role in cell proliferation and apoptosis (22). Its upregulation in non-responders may indicate an increased resistance to treatment-induced cell death (23), thereby reducing the efficacy of immunotherapy and chemotherapy. This suggests that targeting miR-99b-3p could potentially enhance the sensitivity of cancer cells to treatment. For instance, Chang et al. found that miR-99b-3p is induced by vitamin D3 and contributes to its antiproliferative effects in gastric cancer cells by targeting HOXD3 (24). MiR-320d has been implicated in the regulation of the cell cycle and cell differentiation (25). The upregulation of miR-320d in non-responders may lead to altered cell cycle progression, making cancer cells less susceptible to the cytotoxic effects of chemotherapy. This highlights the potential of miR-320d as a target for combination therapies aimed at overcoming treatment resistance. Lv et al. reported that exosomal long non-coding RNA LINC00662 promotes non-small cell lung cancer progression by the miR-320d/E2F1 axis (26). MiR-100-5p is involved in the regulation of the PI3K/AKT signaling pathway (27), which is crucial for cell survival and growth (28). The upregulation of miR-100-5p in non-responders may activate this pathway, promoting cancer cell survival and proliferation. Targeting miR-100-5p could therefore be a promising strategy to enhance the effectiveness of immunotherapy and chemotherapy. Zhou et al. found that HIF1A activates the transcription of lncRNA RAET1K to modulate hypoxia-induced glycolysis in hepatocellular carcinoma cells via miR-100-5p (29). MiR-193a-5p has been associated with cancer metastasis and invasion (30). Its upregulation in non-responders may indicate a more aggressive tumor phenotype, which is less responsive to treatment. This suggests that miR-193a-5p could be a potential target for therapies aimed at inhibiting cancer progression and improving treatment outcomes. Yu et al. explored the mechanisms of miR-193a-5p in regulating lung cancer metastasis by downregulating the ERBB4/PIK3R3/mTOR/S6K2 signaling pathway (31). Understanding these mechanisms can inform the development of targeted therapies or combination strategies to improve treatment outcomes in lung adenocarcinoma. For example, inhibitors of these upregulated miRNAs could be developed to enhance the efficacy of immunotherapy and chemotherapy. Additionally, combining miRNA-targeted therapies with existing treatment modalities may help overcome resistance and improve patient prognosis. Further research is needed to elucidate the specific molecular mechanisms and validate these miRNAs as potential therapeutic targets.

To the best of our knowledge, this is the first study suggesting that sEV-derived miRNAs can be used to identify true responders to checkpoint blockade combined with chemotherapy. This study is also the first to reveal that predictive models based on sEV miRNA combinations have a good performance in identifying populations of responders to immunotherapy combined with chemotherapy. No previous reports are available on the six sEV-derived miRNAs used to construct the models regarding their expression and association with the clinical treatment response. Previous studies have indicated that miR-193a-5p might play a critical role in cancer initiation and development (32). Yu et al. explored the underlying mechanisms of miR-193a-5p in regulating lung cancer metastasis (31). miR-99b-3p plays an anti-proliferative role in gastric cancer cells by targeting HoxD3 (24). Jakob et al. found that miR-99b-3p was positively correlated with improved survival in oral squamous cell carcinoma patients (33, 34). LINC00662 promotes the progression of non-small cell lung cancer (NSCLC) via the miR-320d/E2F1 axis (26). In 2020, Peng et al. reported that miR-320d was obviously increased in PD groups of NSCLC patients after immunotherapy (35). Zhu et al. found that miR-320d inhibited the progression of EGFR-positive colorectal cancer and might be a promising therapeutic target (36). The expression of miR-6815-5p in lung adenocarcinoma is significantly higher than that in normal tissue (37). Liu et al. found that lung adenocarcinoma progression was facilitated by miR-96-5p via ARHGAP6 (38). In 2022, Wu et al. identified that the Wnt signaling pathway was regulated by the circFBXO7/miR-96-5p/MTSS1 axis in ovarian cancer (39). Moreover, the role of miR-96-5p as a target for mediating sunitinib resistance in clear cell renal cell carcinoma has also been reported (40). A previous study indicated that the downregulation of exosomal miR-100-5p was associated with cisplatin resistance in A549 cells (41). Lai et al. found that miR-100-5p was a potential therapeutic target in drug-resistant NSCLC (42). In hepatocellular carcinoma cells, miR-100-5p plays a critical role in hypoxia-induced glycolysis (29). Moreover, miR-100-5p promotes the progression of papillary thyroid cancer by targeting FZD8 (43). The role of miR-100-5p as a potential biomarker for the diagnosis of breast carcinoma has also been reported (44).

This study had some limitations. First, more patients should be recruited for prospective clinical validation with a large sample size prior to the model’s application in the clinic. To address this, we plan to initiate a multicenter trial to validate the sEV miRNA-based model across diverse populations, ensuring robustness and generalizability. Second, future exploration of the regulatory mechanisms should also consider the validation in cell experiments *in vitro* and animal experiments *in vivo*. For instance, organoid models and patient-derived xenografts could elucidate how these miRNAs modulate immune-chemotherapy resistance. Third, we chose only six plasma sEV miRNAs as the most promising biomarkers for identifying responders to immunotherapy combined with chemotherapy, and future studies should employ high-throughput sequencing to screen additional miRNAs and integrate multi-omics data (e.g., proteomics, metabolomics) to enhance predictive accuracy.

To translate these findings from bench to bedside, standardized protocols for sEV isolation and miRNA quantification must be established, potentially through collaborations with diagnostic companies. Additionally, developing point-of-care devices for rapid sEV miRNA detection could facilitate real-time clinical decision-making. Importantly, the sEV miRNA strategy holds potential as a pan-cancer diagnostic approach. For example, similar methodologies could be applied to gastrointestinal or breast cancers, where immunotherapy efficacy also varies widely. Comparative studies across cancer types may reveal conserved miRNA signatures or tissue-specific biomarkers, further advancing precision oncology.

In conclusion, we explored the potential of plasma sEV miRNAs as biomarkers for identifying true responders to checkpoint blockade combined with chemotherapy. After profiling the comprehensive expression of sEV miRNAs in lung adenocarcinoma patients, six differentially expressed sEV miRNAs were detected. Interestingly, all of these sEV miRNAs exhibited good identification ability for distinguishing responders to checkpoint blockade combined with chemotherapy. Moreover, multiple biological signaling pathways involved during this process were also investigated. The results suggest that plasma sEV miRNAs are promising non-invasive biomarkers for identifying true responders to immunotherapy combined with chemotherapy.

## Data Availability

The datasets presented in this study can be found in online repositories. The names of the repository/repositories and accession number(s) can be found in the article/**Supplementary Material**.
