# Supplementary material for: Small extracellular vesicle miRNAs as biomarkers for predicting antitumor efficacy in lung adenocarcinoma treated with chemotherapy and checkpoint blockade
Source: Front Immunol. 2025 Mar 31;16:1573043. doi: 10.3389/fimmu.2025.1573043 (PMC11994727; doi:10.3389/fimmu.2025.1573043)
Supplement: Supplementary file 1 [file DataSheet1.docx]

**Supplementary Material**

**
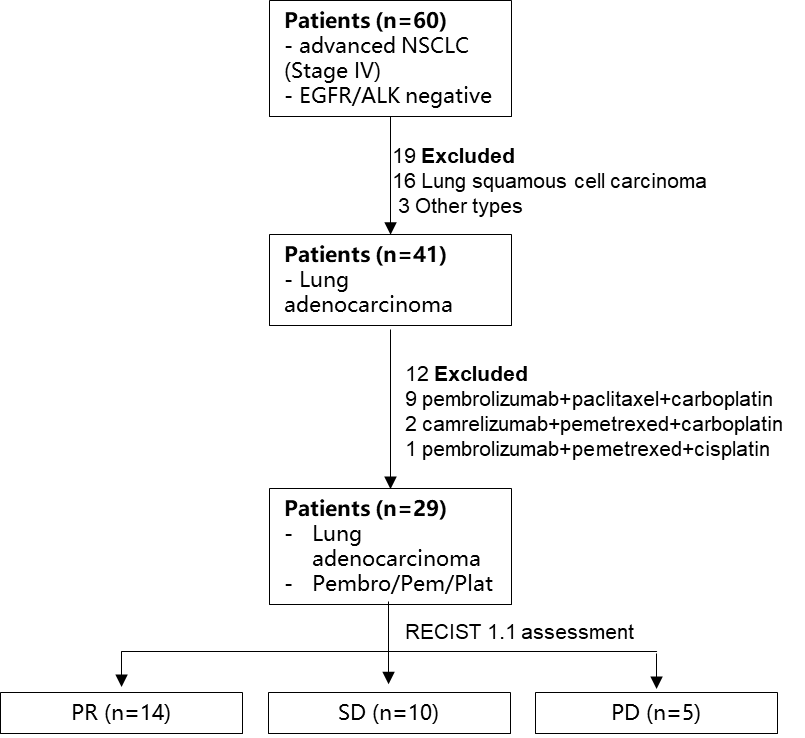
**

**Figure S1. Patient flow diagram.**


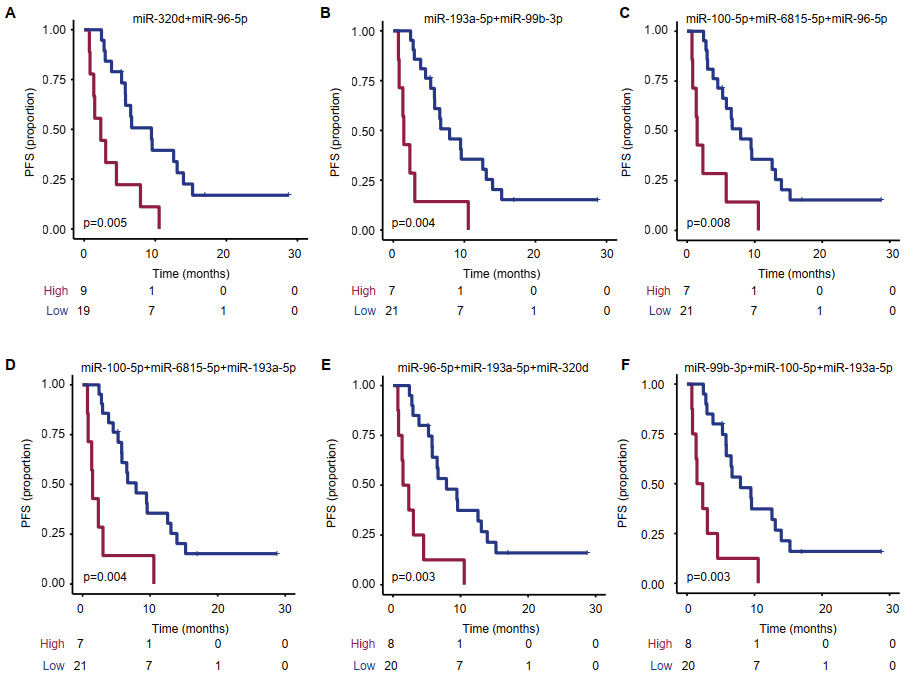


**Figure S2. PFS survival analysis.**

**(A–F)** PFS survival curves based on the sEV miRNA combinations of miR-320d and miR-96-5p **(A)**, miR-193a-5p and miR-99b-3p **(B)**, miR-100-5p, miR-6815-5p, and miR-96-5p **(C)**, miR-100-5p, miR-6815-5p, and miR-193a-5p **(D)**, miR-96-5p, miR-193a-5p, and miR-320d **(E)**, miR-99b-3p, miR-100-5p, and miR-193a-5p **(F)**.


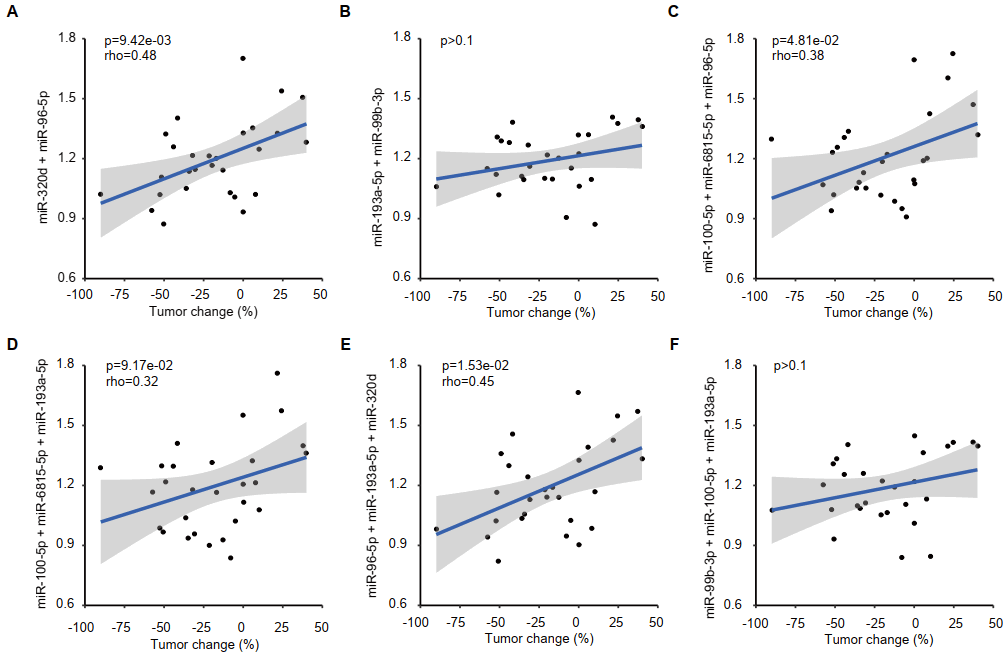


**Figure S3. Correlation between risk score and tumor changes.**

**(A)** Correlation analysis between tumor change (%) and the combination of miR-320d and miR-96-5p. **(B)** Correlation analysis between tumor change (%) and the combination of miR-193a-5p and miR-99b-3p. **(C)** Correlation analysis between tumor change (%) and the combination of miR-100-5p, miR-6815-5p, and miR-96-5p. **(D)** Correlation analysis between tumor change (%) and the combination of miR-100-5p, miR-6815-5p, and miR-193a-5p. **(E)** Correlation analysis between tumor change (%) and the combination of miR-96-5p, miR-193a-5p, and miR-320d. **(F)** Correlation analysis between tumor change (%) and the combination of miR-99b-3p, miR-100-5p, and miR-193a-5p.


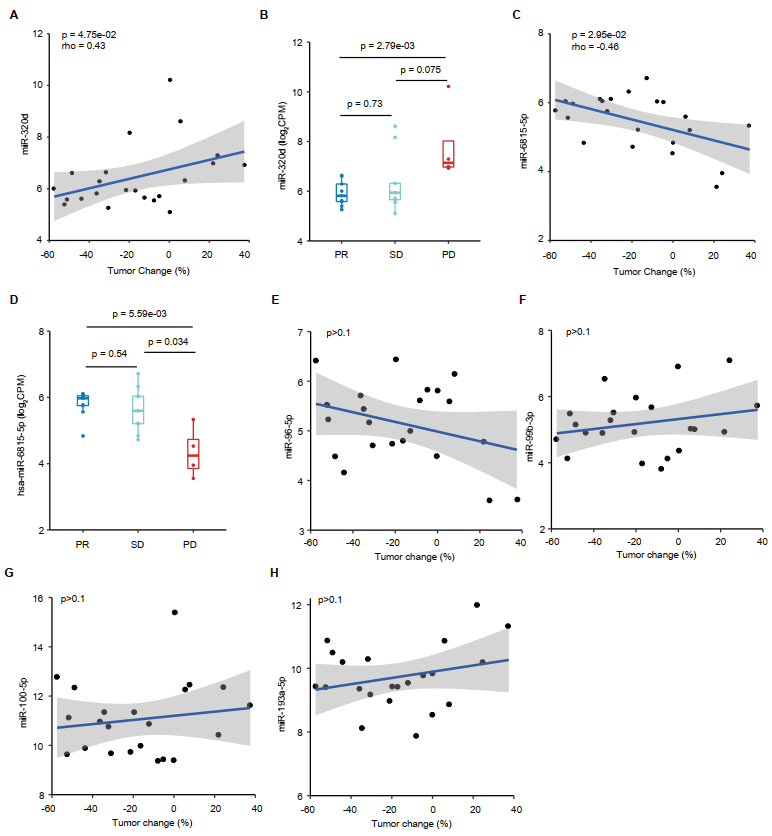


**Figure S4. Correlation between sEV miRNA expression and tumor change.**

**(A)** Correlation analysis between miR-320d and the percentage of tumor change. **(B)** Analysis of the expression levels of miR-320d in PR, SD, and PD samples. **(C)** Correlation analysis between miR-6815-5p and the percentage of tumor change. **(D)** Expression levels of miR-6815-5p in PR, SD, and PD samples. **(E-H)** Correlation analysis between the percentage of tumor change and miR-96-5p (E), miR-99b-3p **(F)**, miR-100-5p **(G)**, and miR-193a-5p **(H)**.


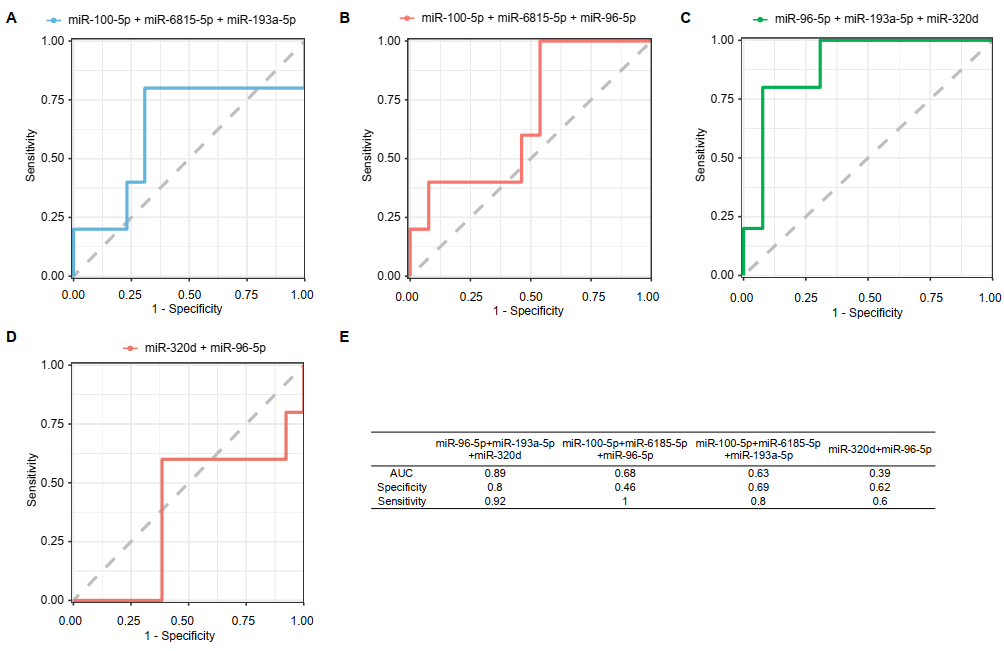


**Figure S5. Screening for the optimal miRNA combination based on qRT-PCR verification.**

**(A–F)** The best miRNAs with the highest AUC values based on the random combination of two or three miRNAs among the six miRNAs.

| **Categories** | **Number (%)** |
| --- | --- |
| **Gender** |  |
| Male | 23(79%) |
| Female | 6 (21%) |
| **Age** |  |
| > 50 | 25 (86%) |
| ≤ 50 | 4(14%) |
| **Smoking history** |  |
| Never | 10 (34%) |
| Ever | 19(66%) |
| **Histology** |  |
| Lung adenocarcinoma | 29 (100%) |
| lung squamous cell carcinoma | 0 (0%) |
| **ECOG PS** |  |
| 0 | 0 (0%) |
| 1 | 29 (100%) |
| 2 | 0 (0%) |
| **Stage** |  |
| I-II | 0 (0%) |
| III-IV | 29(100%) |
| **Drug** |  |
| pembrolizumab combined with peme | 29 (100%) |
| Others | 0(0%) |
| **Efficacy evaluation** |  |
| PR | 14(48%) |
| SD | 10(35%) |
| PD | 5(17%) |

**Table S1. Clinical characteristics of patients.**

| **miRNA** | **Stem Loop RT Primer** | **Forward Primer** | **Probe** |
| --- | --- | --- | --- |
| miR-451a | GTCGTATCCAGTGCAGGGTCCGAGGACTGGATACGACAACTCA | GCGCGAAACCGTTACCATTACTG | CTGGATACGACAACTCAG |
| miR-96-5p | GTCGTATCCAGTGCAGGGTCCGAGGACTGGATACGACAGCAAA | GCGCGTTTGGCACTAGCACATTT | CTGGATACGACAGCAAA |
| miR-193a-5p | GTCGTATCCAGTGCAGGGTCCGAGGACTGGATACGACTCATCT | TGGGTCTTTGCGGGCGA | CTGGATACGACTCATCTCG |
| miR-100-5p | GTCGTATCCAGTGCAGGGTCCGAGGACTGGATACGACCACAAG | TCTCTAACCCGTAGATCCGAAC | CTGGATACGACCACAAG |
| miR-320d | GTCGTATCCAGTGCAGGGTCCGAGGACTGGATACGACTCCTCT | GCGCGAAAAGCTGGGTTGAGA | CTGGATACGACTCCTCT |
| miR-6815-5p | GTCGTATCCAGTGCAGGGTCCGAGGACTGGATACGACAATGAC | GCGCGTAGGTGGCGCCGGAGGA | CTGGATACGACAATGACTC |
| miR-99b-3p | CTCAACTGGTGTCGTGGAGTCGGCAATTCAGTTGAGCCTGGATACGACCGGACC | GCCGAGCAAGCTCGTGTCTGT | ATACGACCGGACCCA |
| universal reverse primer | TGCAGGGTCCGAGG/CTCAACTGGTGTCGTGGA | | |

**Table S2. The primer sequences used for qRT-PCR.**

| **miRNA** | **Risk score model** |
| --- | --- |
| miR-320d + miR-96-5p | Risk score = 1.3667 + 0.1051* (miR-320d) – 0.1641* (miR-96-5p) |
| miR-193a-5p + miR-99b-3p | Risk score = -0.1994 + 0.1122* (miR-193a-5p) + 0.0590* (miR-99b-3p) |
| miR-100-5p + miR-6815-5p + miR-96-5p | Risk score = 2.2004 + 0.0573* (miR-100-5p) – 0.1826* (miR-6815-5p) – 0.1230* (miR-96-5p) |
| miR-100-5p + miR-6815-5p + miR-193a-5p | Risk score = 0.8594 + 0.0397 * (miR-100-5p) + 0.0966 * (miR-6815-5p) – 0.1917* (miR-193a-5p) |
| miR-96-5p + miR-193a-5p + miR-320d | Risk score = 0.9107 + 0.0510 * (miR-193a-5p) + 0.0943 * (miR-320d) –0.1584* (miR-96-5p) |
| miR-99b-3p + miR-100-5p + miR-193a-5p | Risk score = –0.6055 + 0.1197 * (miR-193a-5p) + 0.0311 * (miR-100-5p) + 0.0564 * (miR-99b-3p) |

**Table S3. The miRNAs and coefficients used for the construction of the prediction models.**

| miRNA | Specificity | Sensitivity | AUC |
| --- | --- | --- | --- |
| miR-320d + miR-96-5p | 79.20% | 100.00% | 92.50% |
| miR-193a-5p + miR-99b-3p | 87.50% | 100.00% | 92.50% |
| miR-100-5p + miR-6815-5p + miR-96-5p | 87.50% | 100.00% | 97.50% |
| miR-100-5p + miR-6815-5p + miR-193a-5p | 91.70% | 100.00% | 97.50% |
| miR-96-5p + miR-193a-5p + miR-320d | 83.30% | 100.00% | 95.00% |
| miR-99b-3p + miR-100-5p + miR-193a-5p | 91.70% | 100.00% | 94.20% |

**Table S4. Performance of the ROC curve in different prediction models.**
